# Therapy objectives, treatments modalities and outcomes used by physiotherapists for adolescent idiopathic scoliosis in Alberta, Canada

**DOI:** 10.1186/1748-7161-8-S1-O14

**Published:** 2013-06-03

**Authors:** EC Parent, D Buyks, J Clough, L Jespersen, J Gibson, J Lessard

**Affiliations:** 1 University of Alberta, Department of Physical Therapy, Faculty of Rehabilitation Medicine, Edmonton, Canada; 2 Alberta Health Services, Glenrose Rehabilitation Hospital, Edmonton, Canada

## Background

Progression of scoliosis may lead to self-esteem issues, pain, respiratory complications and limited function. There is a paucity of information regarding how physical therapists manage adolescent idiopathic scoliosis (AIS).

## Aim

The aim of this study was to determine the objectives, treatments and outcomes addressed by physiotherapists in the non-operative management of AIS.

## Methods

A web survey was emailed to 1599 outpatient physiotherapists in Alberta identified from the College of Physical Therapists of Alberta’s registry in 2009. The 30 question survey was adapted from previous back pain studies [1], and collected information regarding demographics, typical case load, experience and the objectives, treatments, and outcomes used in the treatment of AIS. Responses from subgroups of therapists based on experiences (<=10, >10-20, >=20 years) and based on work settings (rural or urban) were compared using Chi-square tests. A retrospective review of the Edmonton scoliosis clinic charts (2000-08) was used to estimate the proportion of patients with AIS referred to physiotherapy.

## Results

Only 15% of all patients with AIS were referred to physiotherapy with a mean age of 16 ± 3 years, and a mean Cobb angle of 26 ± 15 degrees. The response rate from therapist was 11.9%,after 2 reminders with valid responses from 147 therapists. The top objectives pursued by physiotherapists in Alberta were pain reduction (80%), stopping curve progression (57%) and improving body image (45%). Therapists with 0-10 years of experience ranked pain reduction and body image improvements significantly higher than therapists with 10-20 or over 20 years of experience. No therapist reported using scoliosis specific exercises. Stabilization exercises (76%), non-scoliosis specific postural approaches (73%), and mobilizations (55%) were the highest ranked treatment methods used, with mobilizations being used significantly more frequently in rural settings. The primary outcomes documented by physical therapists in Alberta were pain (75%), subjective posture observation (73%), and range of motion (69%).

## Conclusion

The objectives, treatments, and outcomes pursued by Alberta’s physiotherapists while managing AIS are variable, depending on experience and practice settings. Alberta practices did not fully match published recommendations supporting quality of life as primary therapy objective [2] and the use of scoliosis-specific approaches [3].
